# Large-Scale Cortical Functional Organization and Speech Perception across the Lifespan

**DOI:** 10.1371/journal.pone.0016510

**Published:** 2011-01-31

**Authors:** John P. Sheppard, Ji-Ping Wang, Patrick C. M. Wong

**Affiliations:** 1 The Roxelyn and Richard Pepper Department of Communication Sciences and Disorders, Northwestern University, Evanston, Illinois, United States of America; 2 Department of Biomedical Engineering, Northwestern University, Evanston, Illinois, United States of America; 3 Department of Statistics, Northwestern University, Evanston, Illinois, United States of America; 4 Department of Otolaryngology—Head and Neck Surgery, Northwestern University, Chicago, Illinois, United States of America; Indiana University, United States of America

## Abstract

Aging is accompanied by substantial changes in brain function, including functional reorganization of large-scale brain networks. Such differences in network architecture have been reported both at rest and during cognitive task performance, but an open question is whether these age-related differences show task-dependent effects or represent only task-independent changes attributable to a common factor (i.e., underlying physiological decline). To address this question, we used graph theoretic analysis to construct weighted cortical functional networks from hemodynamic (functional MRI) responses in 12 younger and 12 older adults during a speech perception task performed in both quiet and noisy listening conditions. Functional networks were constructed for each subject and listening condition based on inter-regional correlations of the fMRI signal among 66 cortical regions, and network measures of global and local efficiency were computed. Across listening conditions, older adult networks showed significantly decreased global (but not local) efficiency relative to younger adults after normalizing measures to surrogate random networks. Although listening condition produced no main effects on whole-cortex network organization, a significant age group x listening condition interaction was observed. Additionally, an exploratory analysis of regional effects uncovered age-related declines in both global and local efficiency concentrated exclusively in auditory areas (bilateral superior and middle temporal cortex), further suggestive of specificity to the speech perception tasks. Global efficiency also correlated positively with mean cortical thickness across all subjects, establishing gross cortical atrophy as a task-independent contributor to age-related differences in functional organization. Together, our findings provide evidence of age-related disruptions in cortical functional network organization during speech perception tasks, and suggest that although task-independent effects such as cortical atrophy clearly underlie age-related changes in cortical functional organization, age-related differences also demonstrate sensitivity to task domains.

## Introduction

Aging is characterized by marked declines in sensory and cognitive functions [1]–[4], and a vast literature implicates such age-related changes to co-occur not only with differences in functionally localized cortical activity [5]–[8], but additionally in disrupted functional interactions spanning distributed, complex brain networks [9]–[12]. Given these widespread changes, large-scale methods that consider functional organization across the entire cortex become critical to fully explore age-related differences in brain function that underlie sensory and cognitive processes.

In recent years, graph theoretic analysis has offered a powerful data-driven framework to explore the topological organization of brain networks [13]–[16]. Previous studies have established that brain structural and functional networks maintain a small-world organization optimized for both high local and global efficiency of information transfer [17]–[19]. This small-world organization balances opposing demands for segregated (localized) and integrated (distributed) processing, both hypothesized to be crucial for higher-level cognition [20]–[22]. Differences in these small-world properties have been associated with various neurological disorders [23], brain trauma [24], and even intelligence [25]. Studies have also reported changes in brain topological organization over the course of development and in senescence [26]–[30], implying that the brain undergoes dynamic functional reorganization across the lifespan. In particular, recent findings by Achard & Bullmore (2007) and Wang et al. (2010) indicate reduced efficiency of global information transfer in older adult networks during both rest and memory task performance [26], [29], suggesting that age-related cognitive deficits could be associated with declines in efficient small-world organization.

These functional differences are underlain by neuroanatomical changes across the lifespan. Such changes include widespread atrophy of both subcortical and cortical grey matter structures [31]–[34], atrophy and demyelination of white matter fiber tracts [12], [35], [36], and changes in neurochemistry [37]. Findings of reductions in long-range axonal connections have led to the hypothesis that age-related cognitive decline may arise from structural disconnections [36]. More recently, diffusion tensor imaging has also revealed disrupted small-world organization in anatomical connectivity networks of older adults [38]. Presumably, these age-related anatomical differences should also be associated with disruptions in functional network organization, but to our knowledge, such associations have yet to be reported.

Given these pervasive, co-occurring functional and neuroanatomial changes, the question also arises whether age-related effects on brain functional organization are independent of cognitive domains (i.e., reflecting task-independent physiological declines), or show task specificity. Recently, Wang et al. examined changes in functional networks of younger and older adults obtained via fMRI during memory encoding and retrieval tasks involving visually presented words and pictures [26] (see [39] for original experiment by Grady et al.). Observing consistent age-related changes in network topology across task states, the authors argued that age-related network reorganization derives from a common biological factor rather than reflecting specificity to particular cognitive tasks, building upon previous “common cause” hypotheses of aging (see [39]–[41]). These consistent age-related differences have been suggested to arise from decreased ability to inhibit default-mode areas (regions that normally show decreased activity during task performance) coupled with reduced ability to activate cognitive areas such as the dorsolateral prefrontal cortex [39]. Nevertheless, it remains possible that age-related changes in network organization may differ under more diverse task states, particularly those involving other sensory modalities. As far as we are aware, however, no other graph theoretic studies of age-related effects on task-related functional networks have been reported, so whether these age-related disruptions in functional network organization are truly task-independent or show task-dependent effects remains uncertain.

To address these questions, we employed functional MRI to investigate age-related differences in large-scale cortical networks associated with speech perception tasks. In the scanner experiment, we tested 12 younger and 12 older adults' abilities to recognize spoken word stimuli and match them to objects on a screen (given a choice of three alternative pictures) in both a quiet listening condition and in loud multi-talker background noise [8]. Speech perception is a unique paradigm to examine task-dependence of these network properties as it requires integrated function between both auditory-sensory and general cognitive brain regions, which form a distributed spoken language network [42]. Despite the brain's capacity for language, perceiving speech in high levels of noise can be very difficult even for younger adults with normal hearing abilities. Hearing in noisy environments is particularly challenging for older adults, and while much past work has considered the peripheral auditory system's contributions to age-related hearing decline [43], [44], converging evidence implicates sensory deficits in the central nervous system, including the cerebral cortex [45]–[47]. By testing spoken word perception in both quiet and loud noise, this experimental design allowed for comparisons during a condition eliciting comparable (near-perfect) behavioral performance in the younger and older adults (i.e., speech perception in quiet), and a behaviorally very demanding condition (i.e., speech perception in loud noise) exhibiting significantly reduced performance in the older adults relative to the younger subjects.

In the present analysis, we applied graph theoretic analysis to construct weighted cortical functional networks for each subject and listening condition based upon interregional correlations of the fMRI signal among 66 cortical regions. Graph measures describing the efficiency of global and local information transfer were computed within each individual network node (i.e., *regional* measures) and also averaged across all nodes in each network (i.e., *whole-cortex* measures) to describe the overall network topology. We compared both regional and whole-cortex measures across age groups and task conditions, after normalizing the graph measures to values in surrogate random networks. Additionally, to test for a relationship between age-related changes in functional network properties and neuroanatomical characteristics, we examined the relationship between whole-cortex network measures and mean cortical thickness.

Given previous evidence of age-related disruptions in resting-state and memory task-related networks, we expected to observe decreased global efficiency within older adult networks across both the quiet and noisy listening conditions. However, we hypothesized that these differences should arise from regional disruptions in areas implicated in speech perception rather than reflecting only task-independent changes. Previous analysis of these data uncovered reduced activation in bilateral posterior superior temporal gyrus (i.e., auditory cortex) of older adults coupled with age-related increases in cognitive areas in prefrontal and posterior parietal cortex, changes believed to underlie a compensatory mechanism for reduced sensory abilities in aging [8], [32]. Therefore, we expected that age-related disruptions in functional organization would involve this set of regions. Furthermore, comparison of fMRI responses within younger adults revealed increased activation during the noisy (relative to the quiet) listening condition, involving distributed frontal and temporal areas as well as bilateral precuneus [48]. We thus also expected to observe regional changes in network organization across task conditions.

To foreshadow the results, we found significant age-related decreases in global (but not local) network efficiency, agreeing with previous studies of age effects on large-scale brain networks [26], [29]. Regional decreases in both global and local efficiency were also identified, and were localized nearly exclusively to bilateral auditory areas in the temporal cortex. We found no main effects of task condition on whole-cortex network properties, but regional task-related changes were identified in several areas, most prominently involving frontal and parietal cortex. In addition, a significant correlation between global network efficiency and mean cortical thickness was found across all subjects. Crucially, we uncovered a significant group x listening condition interaction effect for local efficiency at the whole-cortex level, which argues that age-related changes in brain functional organization are not task-independent but rather interact with specific sensory stimuli or behavioral states. Overall, this study provides a first report on age-related differences in large-scale cortical network organization underlying speech perception, and argues that although age-related changes in whole-cortex network topology are found consistently across a diversity of task conditions, they also exhibit task specificity.

## Results

### Cortical network construction

**Figure 1:**
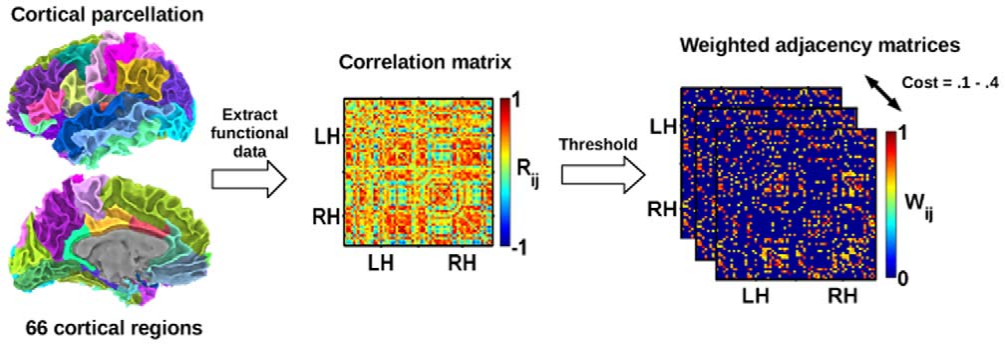
Construction of weighted cortical functional networks. Using Freesurfer, cortical surface maps were prepared for each subject and parcellated into an atlas of 66 cortical regions. Correlation matrices were generated for each subject and listening condition by computing Pearson's correlations between fMRI activation levels across each pair of regions. Undirected functional networks were obtained by applying absolute thresholds to the correlation matrices to obtain networks over a range of costs, with functional weights assigned based on the (absolute) correlation between connected regions. LH/RH: Left/right hemispheres.

We constructed weighted functional networks for each subject (separately for each listening condition) by assigning connections based on the inter-regional correlation of fMRI responses between 66 cortical regions specified in the Desikan-Killiany atlas (see Table 1 for list of regions with abbreviations), after projecting subjects' functional data onto cortical surface maps using Freesurfer and SUMA software [49]–[51]. Weighted functional connections were assigned when the (absolute) Pearson's correlation of the fMRI signals between regions exceeded specified thresholds, leading to networks with fixed costs (i.e., the fraction of existing connections in the network) in the range of 0.1–0.4 (increments of 0.02). This allowed comparisons to be controlled for the number of nodes and edges in each network. Figure 1 provides a schematic outlining this process. While some previous studies have used binary (e.g., assigning equal weight to all connections) as opposed to weighted networks, weighted networks provide a more realistic depiction of brain connectivity by taking into account not only the organization of connections but the magnitude of functional connection strengths between regions, possibly offering greater sensitivity to differences in network structure [24], [52]. Following network construction, measures of global (*E_glob_*) and local (*E_loc_*) efficiency were computed and averaged across all cortical regions to quantify the efficiency of global and local information transfer within the networks. (See Materials and Methods for further details on network construction and for descriptions of the network measures.)

**Table 1:** List of anatomical regions comprising the cortical functional networks.

Bank of the superior temporal sulcus - BSTS	Pericalcarine – PERI
Caudal anterior cingulate - CAC	Postcentral gyrus - PSTC
Caudal middle frontal - CMF	Posterior cingulate - PC
Cuneus – CUN	Pars opercularis (Inferior frontal) – POPE
Entorhinal – ENT	Pars orbitalis (Inferior frontal) - PORB
Frontal pole – FP	Pars triangularis (Inferior frontal) – PTRI
Fusiform gyrus – FUSI	Precentral gyrus - PREC
Inferior parietal – IP	Precuneus – PCUN
Inferior temporal – IT	Rostral anterior cingulate - RAC
Isthmus of the cingulate - ISTC	Rostral middle frontal - RMF
Lateral occipital – LOCC	Superior frontal – SF
Lateral orbitofrontal - LOF	Superior parietal - SP
Lingual gyrus – LING	Superior temporal - ST
Medial orbitofrontal - MOF	Supramarginal - SMAR
Middle temporal – MT	Temporal pole – TP
Paracentral lobule - PARC	Transverse temporal - TT
Parahippocampal – PHG	

Cortical anatomical regions were defined based on the Desikan-Killiany atlas [50]. Abbreviations are adopted from [58].

### Age and listening condition effects on whole-cortex network measures

**Figure 2:**
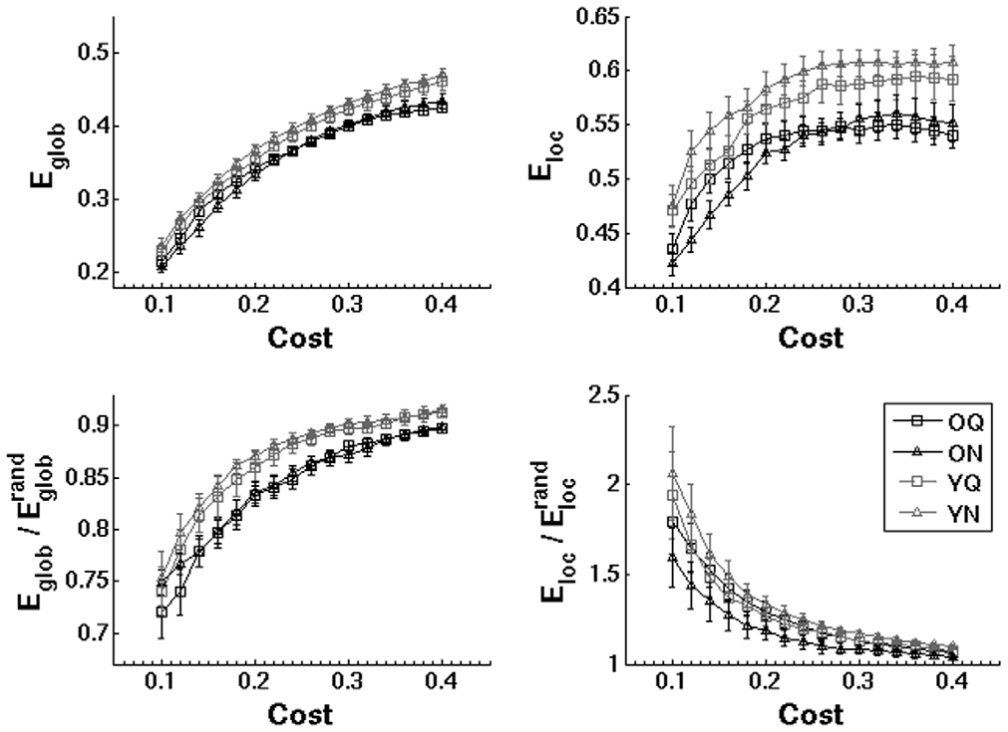
Comparison of weighted global (*E_glob_*) and local (*E_loc_*) efficiency in brain networks. Average network properties are shown for both age groups and listening conditions across the range of network costs (0.1–0.4). To control for individual differences in network connection strengths, normalized global and local efficiency were obtained by comparing network measures to values from randomized surrogate networks (bottom panels). Error bars indicate the standard error of the mean. (O, older adults; Y, younger adults; Q, quiet listening condition; N, noisy listening condition).

Figure 2 displays the average *E_glob_* and *E_loc_* curves across the range of network costs for each subject group and listening condition. Global efficiency increased monotonically as a function of cost, while local efficiency plateaued around a cost of 0.3. Older adults possessed reduced *E_glob_* and *E_loc_* relative to younger adults across the entire cost range, while there were no pronounced differences observed between the quiet and noisy listening conditions. To account for individual differences in mean connection strengths, we computed normalized measures by dividing *E_glob_* and *E_loc_* by the average values obtained in surrogate random networks (bottom panels). Both younger and older adults possessed small-world network topologies characterized by 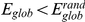 and 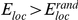, consistent with several previous studies (e.g., [18], [29]). Relative to younger adults, older adults showed consistent reductions in 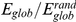, but not in 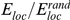. Within each group, 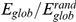 and 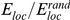 had similar values across listening conditions.

**Figure 3:**
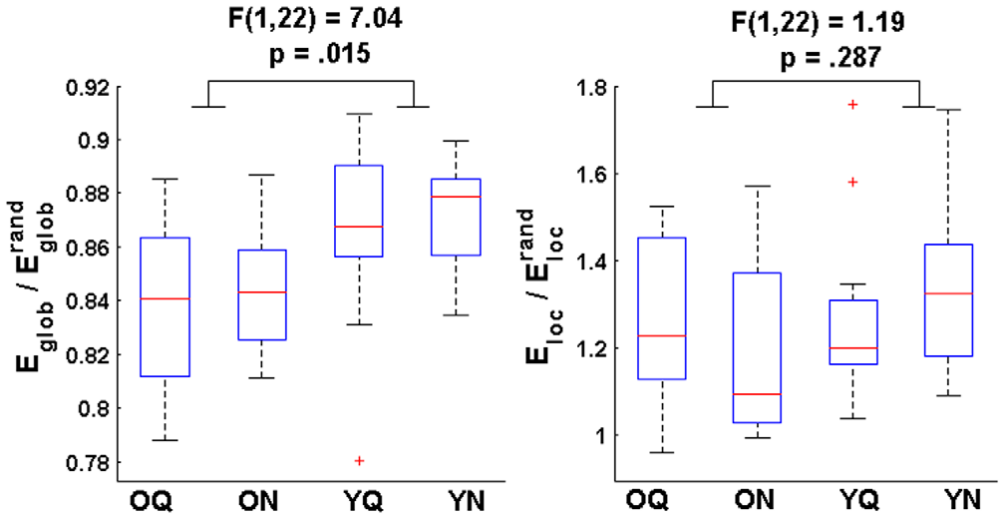
Aging and listening condition effects on whole-cortex network measures. To test for effects of group and listening condition on the normalized weighted graph measures, summary values of 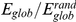 and 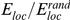 were obtained by averaging the measures across the entire cost curves for each subject and listening condition. A significant effect of group on (cost-averaged) 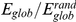 was found (Younger>Older; see left panel). The effect of group on (cost-averaged) 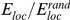 was not significant (right panel). No significant condition or group x condition interaction effects were found. Box plots indicate median, interquartile range, and minimum and maximum values of network measures across all subjects for each age group and listening condition.

To test these differences statistically, summary measures of 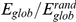 and 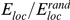 were obtained by averaging the network measures across the entire range of network costs (see Figure 3). Henceforth, we present data on the cost-averaged, normalized network measures. A 2×2 repeated-measures ANOVA revealed a significant effect of group (Younger>Older) on average 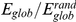 [F(1,22) = 7.04, p = .015]. Although normalized local efficiency was also decreased in the older adults, the main effect of group on 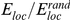 was not significant [F(1,22) = 1.19, p = .287]. There were no significant effects of condition (data not shown), but a significant group by condition interaction was found for 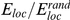 [F(1,22) = 5.98, p = .023]. Figure 3 illustrates the nature of the interaction: younger adult networks exhibited increased 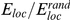 for the noisy listening condition, while the opposite trend was observed in older adults. The group by condition interaction effect for 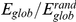 was not significant [F(1,22) = 0.06, p = .809]. These findings corroborate previous functional studies reporting reductions in global efficiency (or increased average path lengths) in older adults during rest and memory task performance [26], [29]. However, contrary to previous reports [26], our results suggest that age-related differences in functional network organization are not task-independent, as the group differences were modulated by the listening condition.

### Head movement analysis

Conceivably, observed differences in brain functional connectivity could be related to head motion in the scanner [53]. However, we found no significant differences in average translational head movement between younger and older adults [t(22) = −1.14, p = .267]. Looking across younger and older adults, we found no significant correlations between head movement and the whole-cortex measures of either 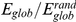 and 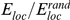 for either listening condition, indicating that the observed group differences were not attributable to subjects' head movement.

### Age and listening condition effects on regional network measures

**Figure 4:**
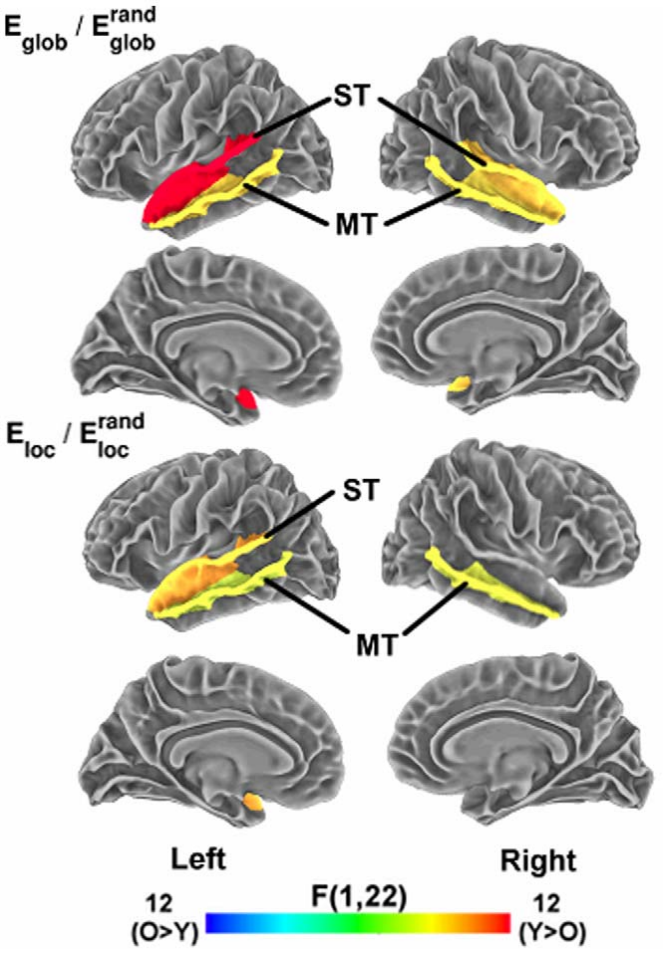
Aging effects on regional network measures. Surface maps display cortical regions showing a significant main effect (p<.05, uncorrected) of age group on regional network measures of 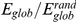 (top panels) and 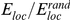 (bottom panels). The color bar indicates direction and magnitude of the main effect of age group on network measures (red: younger>older; blue: older>younger). Abbreviations of cortical regions are provided in Table 1.

**Figure 5:**
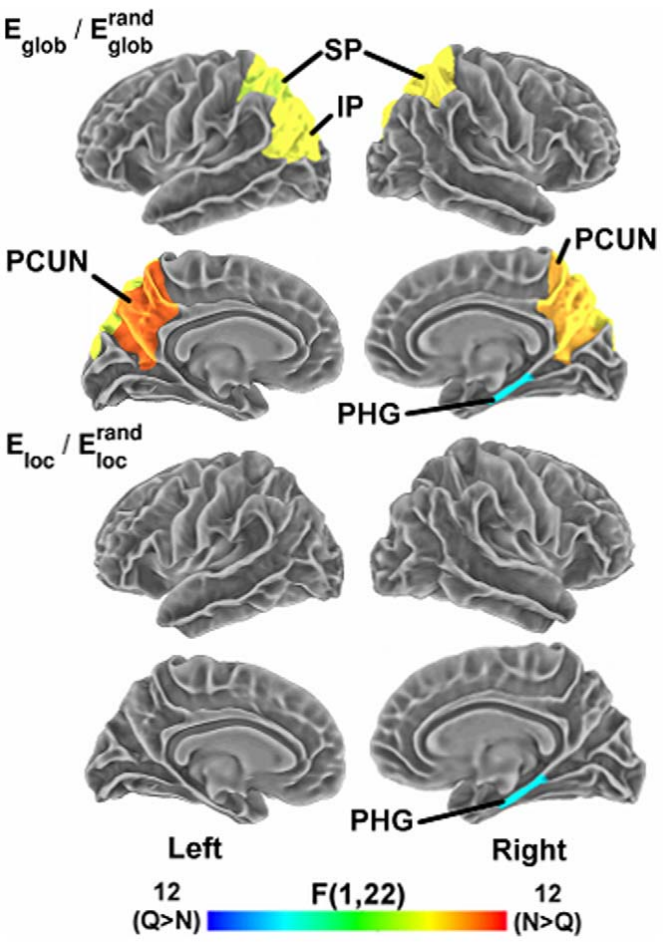
Listening condition effects on regional network measures. Surface maps display cortical regions showing a significant main effect (p<.05, uncorrected) of listening condition (quiet vs. noisy) on regional network measures of 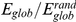 (top panels) and 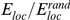 (bottom panels). The color bar indicates direction and magnitude of the main effect of listening condition on network measures (red: noisy>quiet; blue: quiet>noisy).

**Figure 6:**
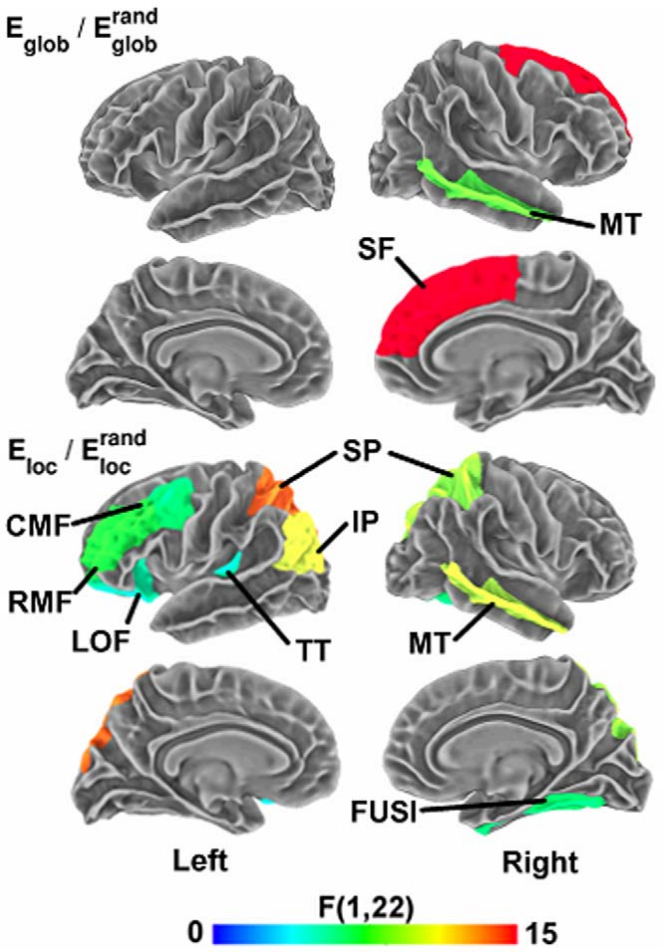
Group x listening condition interaction effects on regional network measures. Surface maps display cortical regions showing a significant interaction effect (p<.05, uncorrected) between age group and listening condition on regional network measures of 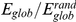 (top panels) and 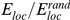 (bottom panels). The color bar indicates the significance (F-statistic) of the interaction effect. Table 2 provides p-values and indicates the nature of the interaction effects in each significant region.

Next, we tested for effects of subject group and listening condition on network measures within individual cortical regions. We applied a single p-value cutoff of 0.05 (uncorrected for multiple comparisons) to establish significance for regional effects; thus, our results at the regional level must be regarded as exploratory. Table 2 lists cortical regions showing significant eff ects of age group and listening condition on regional measures of 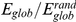 and 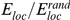. Significant effects of age group, listening condition, and age group x listening condition interaction effects on the network measures are also displayed on cortical surfaces in Figures 4, 5, and 6, respectively (see Table 1 for abbreviations of cortical regions). For 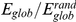, four regions showed decreased values in older adults, localized exclusively in the temporal lobe. These areas included bilateral middle and superior temporal cortex. Age-related decreases in 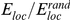 consisted of a subset of three of these temporal areas, with declines in bilateral middle temporal and left superior temporal cortex. These temporal regions are key auditory areas that function in both lower-level acoustic analysis and in sound-to-word recognition [42], [54].

**Table 2:** Effects of age group and listening condition on regional network measures.

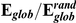				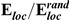			
Group effect	Effect	F(1,22)	Sig.	Group effect	Effect	F(1,22)	Sig.
Left MT	Y>O	6.82	.0159	Left MT	Y>O	4.90	.0376
Left ST	Y>O	13.13	.0015	Left ST	Y>O	8.27	.0088
Right MT	Y>O	6.42	.0189	Right MT	Y>O	5.39	.0299
Right ST	Y>O	7.29	.0131				
				**Condition effect**	**Effect**	**F(1,22)**	**Sig.**
**Condition effect**	**Effect**	**F(1,22)**	**Sig.**	Right PHG	Q>N	6.84	.0158
Left IP	N>Q	5.45	.0291				
Left PCUN	N>Q	9.52	.0054	**Interaction**	**Effect**	**F(1,22)**	**Sig.**
Left SP	N>Q	4.53	.0448	Left CMF	Y(N>Q)>O(N>Q)	6.51	.0182
Right PCUN	N>Q	7.59	.0116	Left IP	Y(N>Q)>O(N>Q)	10.59	.0036
Right SP	N>Q	6.06	.0222	Left LOF	Y(N>Q)>O(N>Q)	5.38	.0300
Right PHG	Q>N	6.81	.0160	Left RMF	Y(N>Q)>O(N>Q)	7.77	.0107
				Left SP	Y(N>Q)>O(N>Q)	13.64	.0013
**Interaction**	**Effect**	**F(1,22)**	**Sig.**	Left TT	Y(N>Q)>O(N>Q)	5.24	.0321
Right MT	Y(N>Q)>O(N>Q)	8.41	.0083	Right FUSI	Y(N>Q)>O(N>Q)	6.51	.0182
Right SF	O(N>Q)>Y(N>Q)	15.03	.0008	Right MT	Y(N>Q)>O(N>Q)	9.83	.0048
				Right SP	Y(N>Q)>O(N>Q)	9.12	.0063

Table lists cortical regions showing significant effects (p<.05, uncorrected) of age group and listening condition on regional measures of 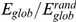 (left) and 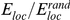 (right). 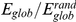 and 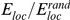 correspond to values averaged over the entire range of network costs (0.1–0.4). Significance levels represent two-tailed p-values obtained from a repeated-measures, mixed-effects ANOVA. See Table 1 for abbreviations of cortical regions.

Additionally, we found several areas that showed main effects of listening condition on the network measures (see Table 2 and Figure 5). The noisy listening condition resulted in increased 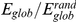 relative to the quiet condition spanning several regions of parietal cortex, including bilateral precuneus, superior parietal, and left inferior parietal cortex, but decreases in the right parahippocampal cortex. Several of these parietal regions (e.g., bilateral precuneus) were previously found to show increased activation during the noisy relative to the quiet listening conditions within the younger adults [48]. In addition, the right parahippocampal cortex exhibited decreased 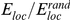 in the noisy condition relative to the quiet condition. Together, several of these regions also overlapped the default mode-network (e.g., precuneus, parahippocampal cortex), a set of regions found to remain active during rest that plays key roles in modulating attentional processes [55]–[57]. These default-mode areas have also been identified as highly connected structural hubs within the cortex [58].

We also observed regions showing an interaction effect between subject group and task condition (see Table 2 and Figure 6). For 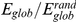, significant group-condition interactions were found only in right middle temporal and right superior frontal cortices. Several regions showed significant group x condition interaction effects for 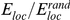, spanning posterior parietal (bilateral superior and left inferior parietal cortex), dorsolateral prefrontal (left rostral and caudal middle frontal cortex), left lateral orbitofrontal cortex, as well as the left primary auditory (transverse temporal), right middle temporal, and right fusiform cortex. The dorsolateral prefrontal cortex (DLPFC) is implicated in working memory as well as higher-level speech processing [59]–[62]. Previously, we found that dorsolateral prefrontal, posterior parietal, left primary auditory, and right middle temporal cortices all showed increased activation during the noisy relative to the quiet listening conditions within the younger adults [48]. Likewise, in nearly all regions showing significant group x condition interaction effects for 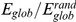 and 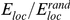, younger adults exhibited greater increases in the noisy relative to the quiet listening condition compared to the older subjects (Table 2).

### Whole-cortex network properties and average cortical thickness

**Figure 7:**
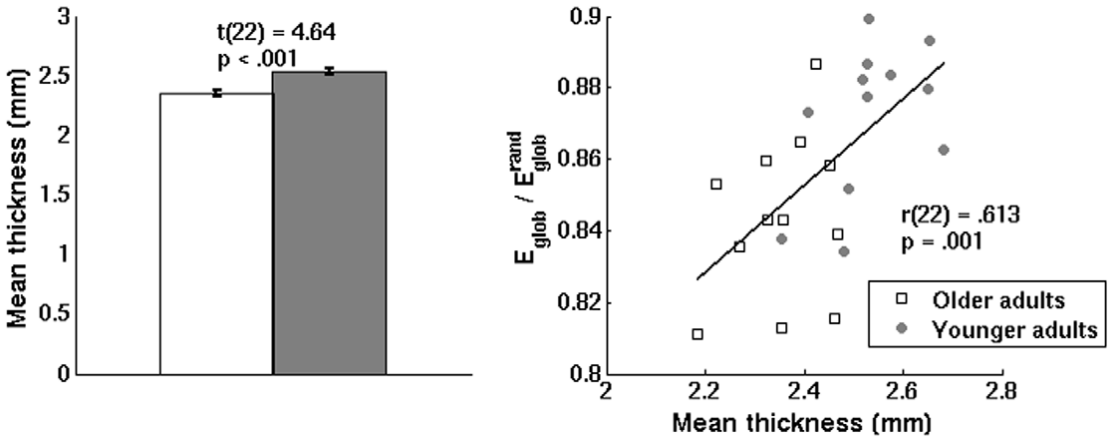
Association between mean cortical thickness and 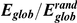. Left panel: Younger adults exhibited increased mean cortical thickness relative to older adults. Error bars indicate the standard error of the mean. Right panel: Across younger and older adults, a significant correlation was found between mean cortical thickness and average 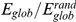. Presented data are from networks obtained from the noisy listening condition.

As anticipated, older adults had significantly reduced mean cortical thickness relative to younger adults (see Figure 7, left panel). We computed Pearson's correlations between cortical thickness and the normalized network measures across all subjects to examine whether gross neuroanatomical atrophy explained the age-related changes in whole-cortex network organization. Mean cortical thickness, 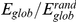, and 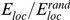 were all normally distributed [p>.05, Shapiro-Wilk tests], and therefore Pearson's correlations were used. Across all subjects, we found a significant positive correlation between mean cortical thickness and 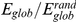 (from networks associated with the noisy listening condition), showing that reduced cortical thickness in older adults was predictive of the observed decreases in global efficiency (see Figure 4, right panel). The correlation between cortical thickness and 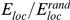 was not significant [r(22) = .348, p = .095]. Although the correlation between cortical thickness and 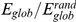 associated with the quiet listening condition did not reach significance [r(22) = .296, p = .160], the similar observed trend suggests that the relationship between cortical atrophy and 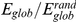 was not task-dependent.

### Analysis of performance (task accuracy) effects

One potential factor influencing results in this study was a significant performance discrepancy between younger and older adults during the noisy listening condition, with reduced task accuracy in the older adults (p<.001) despite normal peripheral hearing abilities [8]. Since individual differences in performance levels and particularly the frequency of errors can impact functional activation patterns during task performance [63], we investigated whether the observed effects on network measures were related to differences in performance (i.e., task accuracy). To test for performance effects, we entered the mean task accuracy for each stimulus block (fraction correct out of four possible trials) as regressors in a general linear model, and repeated the network analysis on the residual functional data. If the observed differences in cortical functional organization were influenced by performance discrepancies, we would expect the results of this residual analysis to differ from the original results.

Interestingly, we observed a different pattern of results in these residual data relative to the original results (see Figures S1, S2 and Tables S1, S2). For 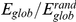, the significant group effect previously observed was lost [F(1,22) = 0.08, p = .786]. Instead, we found a significant condition effect [F(1,22) = 4.99, p = .036], with higher 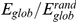 during the noisy listening condition in both subject groups. As in the original analysis, there was no significant group x condition interaction. For 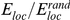, on the other hand, the previously observed group x condition interaction effect was no longer significant [F(1,22) = 0.46, p = .505], but we instead observed a significant group effect [F(1,22) = 4.98, p = .036], with higher 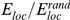 in the younger relative to the older subjects (consistent with the trend in the original results).

The performance effect analysis also revealed a different pattern of results for the regional network measures (Table S2). As in the original analysis, significant regional effects were generally found in temporal, posterior parietal, or prefrontal cortices, areas overlapping a core network of language areas implicated in previous work by us and others [8], [54]. After regressing out performance effects, younger adults showed increases in 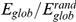 and 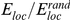 spanning a more extensive, distributed set of regions, including bilateral lateral temporal cortices and extending to prefrontal cortex and regions of the cingulate gyrus (see Table S2). Listening condition effects were also widespread and included temporal, parietal, and frontal cortices. Overall, these results suggest that performance differences indeed influenced the observed results on network measures. However, the significant effects at the whole-cortex and regional levels involving overlapping cortical areas even after regressing out task accuracy effects suggests that task accuracy is only one of multiple factors modulating these age-related differences.

## Discussion

In this study, we applied a weighted graph theoretic analysis to assess cortical functional organization in younger and older adults during performance of a speech perception task in both quiet and noisy listening conditions. We found that older adult cortical networks possessed significantly reduced global (but similar local) efficiency relative to younger adults after normalizing graph measures to values in randomized networks. Most importantly, a significant group x listening condition interaction effect for 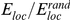 at the whole-cortex level suggested that age-related differences in cortical functional organization are not task-independent, in contrast to previous findings [26]. For both younger and older adults, network measures at the whole-cortex level remained consistent across the quiet and noisy listening conditions. Regionally, older adults showed significantly reduced global and local efficiency concentrated exclusively in auditory and speech areas of the lateral temporal cortex. Regional differences in 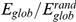 and 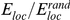 were also found between the two listening conditions (quiet vs. noisy), and group by listening condition interactions were observed in distributed regions spanning posterior parietal, dorsolateral prefrontal, left primary auditory, and right middle and parahippocampal cortices, overlapping key auditory- and working memory-related areas as well as regions of the default-mode network. At the whole-cortex level, 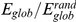 (from the noisy listening condition) correlated positively with mean cortical thickness across all subjects, suggesting that gross neuroanatomical atrophy is associated with declining network efficiency in aging.

### Age- and task-related effects on whole-cortex network properties

This report adds to a body of research linking cognitive aging to disrupted organization of communication pathways encompassing the entire cerebral cortex [12], [26], [30], [64], and extends these findings to task-related functional networks associated with speech perception. Corroborating our findings, Achard & Bullmore (2007) reported decreased global efficiency (and marginally decreased local efficiency) in older adult networks based on resting-state fMRI, and Wang et al. (2010) reported age-related decreases in efficient global (but not local) organization of fMRI networks associated with memory task performance. At the whole-cortex level, the network measures in both our younger and older adults were unaffected by the specific listening condition, agreeing with previous studies reporting consistent global network topology across rest and finger-tapping [65] and across memory encoding and retrieval tasks [26]. However, in contrast to previous studies [26], we found a significant group x condition interaction effect on 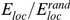, supporting the notion that age-related differences in cortical network organization may be sensitive to task effects.

### Age- and task-related effects on regional cortical network properties

Importantly, the age-related disruptions observed for whole-cortex network properties were underlain by regional changes concentrated in auditory areas of the lateral temporal cortex. Older adults possessed decreased global and local efficiency within bilateral superior and middle temporal cortex. The superior temporal cortices constitute key auditory regions responsible for low-level acoustic analysis; thus, declines in efficient information transfer involving these regions could affect mapping of inputs from the peripheral auditory system onto acoustical representations [42], [54]. As part of the ventral auditory stream, the middle temporal cortex functions in mapping sounds onto word meanings [42], and we previously found a correlation between activation of the right middle temporal gyrus (MTG) and task accuracy in the older adults [8]. In our previous activation analysis, however, age-related reductions in activation during the speech perception tasks were restricted to the posterior superior temporal gyrus (pSTG) [8], whereas the present results uncovered differences extending to additional auditory areas (bilateral middle temporal cortex). On the other hand, regions in prefrontal and posterior parietal cortex did not contain significantly different network properties between groups, even though these regions previously showed increased activation in the older adults. By considering complex interactions within the larger cortical network, this analysis thus provided further insights of age-related functional differences beyond those provided by considering isolated activation patterns.

Additionally, we observed regions that exhibited main effects of listening condition (quiet vs. noisy) on either global or local efficiency. Several of these regions overlapped the default-mode network, including bilateral posterior parietal and right parahippocampal cortex [57], [66], [67]. Since default-mode activation is known to modulate task-oriented states [56], one conceivable explanation for these findings is increased attentional effort during the noisy (more difficult) listening condition.

We also found several cortical areas showing significant group x listening condition interaction effects on the regional network measures. Group x condition interaction effects were observed in left primary auditory cortex, right middle temporal cortex, and in default-mode areas within posterior parietal cortex. Thus, in addition to task-dependent age-related differences on whole-cortex network structure, the two listening conditions evoked differential effects on network organization between younger and older adults at the regional level.

In addition, group x condition interaction effects on 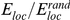 extended to the left dorsolateral prefrontal cortex (DLPFC). The DLPFC participates in a left-lateralized dorsal auditory stream that functions in mapping sounds onto articulatory representations, important in both speech perception and production [42]. DLPFC regions also perform key roles in attention and working memory [60], [61], cognitive functions that facilitate speech perception in both quiet and noise, particularly in older adults [68]. Previously, we found the relative volume of left DLPFC regions to correlate with hearing-in-noise abilities in our older adults [32], indicating that the DLPFC becomes increasingly important for older subjects when perceiving speech in noisy listening environments. Older adults also showed greater increases in DLPFC activation in response to the noisy listening condition compared to younger adults, which we hypothesized to reflect cognitive compensation (i.e., increased reliance on attention and working memory resources) for declining sensory function in aging [8].

### Age-related changes in network structure: Evidence for a common factor?

Based on findings of consistent age-related differences in activation patterns and functional network topology across memory encoding and recognition tasks, Grady et al. and Wang et al. argued that age-related changes in brain network organization derive from a common, domain-general factor [26], [39]. This common factor was hypothesized to involve a declining ability to inhibit task-irrelevant, default-mode activity, coupled with reduced ability to activate cognitive regions such as the DLPFC [26], [39]. Although we also found younger and older adults to show consistent network topologies across different speech perception tasks at the whole-cortex level, we observed a significant group x condition interaction effect on 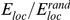, suggesting that age-related differences in cortical functional organization may be task-dependent. Furthermore, in contrast to previous reports of age-related changes concentrated in frontal and parietal regions during memory task performance [26], we observed age-related declines in 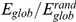 and 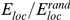 concentrated exclusively in auditory areas of the temporal lobe during speech perception tasks. Our results thus suggest specificity of age-related differences to the auditory nature of the speech perception tasks, and were not indicative of task- or domain-independent changes involving altered default-mode function.

### Age-related effects and cognitive performance

Declining cognitive performance is a central aspect of aging. Even though the older participants in our study possessed normal peripheral hearing abilities, their speech perception performance (i.e., task accuracy) was significantly worse than that of younger adults under the increased sensory/cognitive demands of the noisy listening environment [8]. As individual differences in task performance contribute to differences in functional neuroanatomy [63], performance effects likely are an important contributor to age-related differences in cortical functional organization. Indeed, after regressing out the effects of task accuracy from the imaging data through a residual analysis, we observed different patterns of results from our original analysis. While 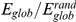 was initially significantly increased in younger adults, we observed a shift in significance towards higher 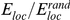 in the younger adults after regressing out performance effects. Conceivably, the observed task performance effects associated with aging may exert a more globally distributed effect on cortical functional organization, while local effects become more pronounced after controlling for performance differences. It is important to note, however, that our findings do not provide evidence of a direct relationship between functional network organization and reduced cognitive performance in aging. Despite age-related differences in task performance, the correlations between task accuracy on the noisy (most difficult) listening condition and 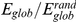 [r(22) = .383, p = .065] and 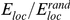 [r(22) = .215, .314] were not significant.

Ultimately, the effects of reduced cognitive performance on cortical functional organization in aging remains an area for further investigation. However, the significant age-related effects at both the whole-cortex and regional levels in this residual analysis suggest that task performance is not the only important factor driving the results observed in this study. These types of complementary analyses may prove useful in future studies to help disentangle the various behavioral, anatomical, and physiological factors influencing functional organization of the cortex in both aging research and comparative neuroimaging studies in general.

### Cortical thickness and whole-cortex network properties

Looking across all subjects, we found a significant positive correlation between mean cortical thickness and 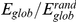, showing that cortical thickness predicted the decrease in 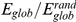 observed across the lifespan. These findings suggest that gross neuroanatomical atrophy (reduced cortical thickness) coincides with less efficient network organization in aging. Neurophysiologically, declining efficiency of global information transfer could potentially result from the loss of neurons and synaptic connections associated with age-related cortical atrophy. However, this observed relationship does not imply causation, and functional differences may also be explained by other physiological factors such as reduced anatomical connectivity arising from atrophy of white matter tracts. Further work is thus needed to clarify the relationship between these various anatomical factors and age-related disruptions in brain functional organization both during rest and cognitive task performance.

### Methodological considerations

It is also worth noting some important limitations of our study. First, similar to previous studies [26], [29], we considered only two groups of younger (19–27 years) and older adults (63–75 years). A more complete picture of age-related changes will require examination of a wider range of ages. Second, our cortical networks were constructed from data acquired from sparse, block-based sampling using a long acquisition time of 14 seconds, and this limits the frequency spectrum of the hemodynamic response able to be observed in the experiment. It is unknown (to our knowledge) whether the age-related changes reported here are also observed across higher frequency bands such as can be assessed using EEG or MEG, although previous MEG research has suggested that network topology remains invariant across a wide range of temporal scales [65]. Previously, there has also been (healthy) debate as to whether correlations of the fMRI signal indicate true synchronization between regions [66], [69] or simply reflect cardiac and respiratory artifacts (e.g., [70]; see also Supplementary Materials of [25]). However, past studies have reported correlations between the hemodynamic response and local field potentials [71], [72], supporting the view that correlations of the fMRI response are indeed indicative of synchronous neural activity. Third, our regional results remain speculatory in nature as we used a liberal p-value threshold of .05 (uncorrected for multiple comparisons). More highly powered studies involving larger cohorts would be informative in confirming these findings. Fourth, one aim of this study was to examine whether age-related changes in functional network organization demonstrated specificity to speech perception tasks. While our paradigm uncovered age-related differences concentrated in auditory areas that differed from studies examining rest [29] and memory task performance [26], cross-study comparisons are limited by variations among cohorts, scanner parameters, and other methodological differences. A comparison of network properties across a more diverse battery of sensory/cognitive tasks within a single cohort of younger and older adults would therefore provide more definitive conclusions [7].

### Conclusion

This study provides evidence of age-related disruptions in large-scale cortical functional organization during performance of a speech perception task. Although the observed age-related differences in whole-cortex network topology partly corroborated previous studies of resting-state connectivity [29] and memory task performance [26], we found a significant group x condition interaction effect on 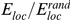, suggesting that age-related differences may show task-dependent effects even at the whole-cortex level. Additionally, we found that age-related differences in 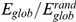 were associated with reduced cortical thickness in the older adults, establishing neuroanatomical atrophy as a task-independent contributor to disrupted cortical functional organization in aging. Though the regional results in our study remain exploratory due to our limited sample size, the observation of age-related differences concentrated exclusively in bilateral auditory areas further suggests task specificity underlying the age-related effects. Listening condition and group x condition interaction effects also overlapped speech- and working memory-related areas in middle temporal, posterior parietal cortex, and the DLPFC, areas known to constitute a core language network [54]. Although converging evidence indicates that disruption of efficient, small-world organization is a hallmark of the aging brain, the present study suggests that task domain-specific factors nevertheless have important effects on cortical network organization.

## Materials and Methods

### Ethics Statement

All subjects in this experiment gave informed written consent prior to inclusion in the study and were compensated monetarily for their participation. All research was conducted under the approval of the Northwestern University Institutional Review Board, and thus adhered to the ethical standards outlined in the 1964 Declaration of Helsinki.

### Overview

In this study, we employed functional magnetic resonance imaging (fMRI) to examine characteristics of cortical functional networks related to speech perception in noise (SPIN) performance in younger (19–27 years) and older (63–75 years) adults [8], [48]. During the scanner experiment, subjects were presented with one-word speech stimuli in both a quiet listening condition and in multi-talker babble that is characteristic of noise in social environments. Simultaneously, subjects were presented with three alternative images on a screen and asked to select the image corresponding to the target word of each stimulus. Subsequently, we applied graph theoretic analysis to explore how properties of the cortical functional networks differed both between younger and older adults and across listening conditions (i.e., quiet versus noisy). We also examined the relationship between network properties and cortical thickness to test whether functional network measures were associated with age-related differences in gross neuroanatomy.

### Subject recruitment

We recruited twelve younger (19–27 years; mean age 21.75±3.05 years (SD); 8 females) and twelve older (63–75 years; mean age = 67.50±3.58 years (SD); 6 females) adults with no reported neurological deficits for participation in the study.

### Speech stimuli

Speech stimuli consisted of a set of twenty target words obtained from [73] that occur with low frequency in American English, and were produced by a Native male speaker (see [48]). Words were then embedded in multi-talker babble noise from the standardized speech perception in noise (SPIN) test at SNR ratios of 20 dB (moderate noise) and −5 dB (loudest noise) [74]. Both the original and noise-embedded stimuli were then RMS amplitude-normalized to a sound pressure level of 65 dB. In the present study, we restrict analysis to the quiet (noise-free) and the loudest (most difficult) noise condition, the latter of which was the only condition to show significant group differences in task accuracy [8].

### Scanner experiment

In the scanner experiment, subjects were presented with one-word speech stimuli via headphones (either in quiet or embedded in multi-talker noise). During each stimulus presentation, subjects were shown three images on a screen, including an image matching the target word and two distracters, and used a response box to select the image corresponding to the target word. For instance, in one trial, subjects heard the word “witch” in the presence of background noise, and had to select between images of a witch and two other objects from the list of stimuli. Stimuli were presented in 12-second blocks consisting of three individual stimuli (each corresponding to the same listening condition), limiting response times to four seconds per stimulus. In total, the fMRI experiment consisted of 60 stimulus blocks per condition as well as 30 null blocks during which no stimuli were presented, with all blocks presented in pseudorandomized order.

### MRI acquisition

MR imaging data were acquired on a Siemens 3T Trio machine at the Center for Advanced MRI at Northwestern University. T1-weighted, high-resolution anatomical images were acquired axially (MP-RAGE; TR/TE = 2300 ms/3.36 ms; flip angle = 9°; TI = 900 ms; matrix size = 256×256; FOV = 22 cm; slice thickness = 1 mm). During the speech perception experiment, T2^*^-weighted functional images were acquired using a susceptibility-weighted EPI pulse sequence (TE = 30 ms; flip angle = 90°; in-plane resolution = 3.4375 mm×3.4375 mm), with 24 slices acquired per scan in an interleaved measurement (3 mm slice thickness; zero gap). Functional image acquisition occurred directly after each 12-second stimulus block and lasted two seconds in duration, resulting in a 14-second repetition time (TR). The sparse functional image acquisition (long TR) ensured there was no scanner noise during stimulus presentation [75], [76]. 210 (60×3+30) 14-second TRs were acquired in total.

### MRI Data Pre-Processing

We pre-processed MRI data using AFNI [77], SUMA [49], and the FreeSurfer analysis suite [51], [78], [79]. Pre-processing of functional time series data was performed in the native three-dimensional (volumetric) space of each individual subject. Initially, the first image from each functional dataset was discarded to minimize T1 equilibration artifacts. Next, differences in the time of acquisition between slices were corrected using Fourier interpolation. Functional data were then corrected for head movement artifacts, using the second image of the acquisition as a reference to which subsequent images were adjusted. Due to the limited frequency spectrum of the acquired functional images (long TR), we did not apply bandpass filtering to the functional data. Subsequently, we mapped the processed volumetric data onto high-resolution, two-dimensional cortical surface meshes prepared for each subject using FreeSurfer and SUMA software, without regressing out the whole-brain signal. To avoid introduction of artificial local correlations in the fMRI signal, no spatial filtering was performed after mapping data onto the cortical surfaces [80]. Further details of these surface-based methods are provided in Methods S1.

Next, using FreeSurfer's automated cortical parcellation [79], we obtained a classification of each subjects' cortical surface into 66 anatomical regions (33 per hemisphere), corresponding to the cortical regions defined in the Desikan-Killiany atlas [50]. These anatomical areas defined the nodes of each cortical network (see Table 1 for list of regions). For each subject and listening condition (i.e., quiet and noisy), node-averaged vectors (*V*) were extracted containing the functional data from the stimulus blocks corresponding to the listening condition of interest, and pair-wise, zero time-lag Pearson's correlations were calculated between nodes such that
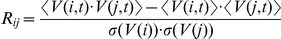
for nodes *i* and *j*, where 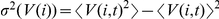 and 〈·〉 indicates temporal averages [81]. This established a raw correlation matrix (*R*) containing the pair-wise correlations of the fMRI responses between each pair of cortical regions for each listening condition.

Subsequently, we defined weighted cortical graphs *G* by assigning undirected, weighted connections (edges) between nodes whose absolute pair-wise correlation exceeded a pre-specified cost threshold, *r_c_*. This resulted in a weighted adjacency matrix (*W*), such that *W_ij_ = *|*R_ij_*| if *|R_ij_|≥r_c_* or *W_ij_ = 0* if *|R_ij_|<r_c_*. The cost thresholds (*r_c_*) were specified separately for each subject in order to generate networks with fixed costs (i.e., the fraction of existing to possible edges in the network). This approach facilitated comparison of network properties by ensuring that each individual network contained the same number of nodes and edges. To ensure that results were not dependent on any particular network cost, we constructed networks over a cost range from 0.10 to 0.40 at increments of 0.02.

### Network measures

Watts & Strogatz originally proposed the characteristic path length (*L_net_*) and cluster coefficient (*C_net_*) to quantify small-world network properties [13]. Alternatively, Latora & Marchiori introduced the efficiency metric, which can quantify the efficiency of both global and local information transfer and generalizes naturally to weighted networks [82]. In an individual node *i*, the global efficiency (*E_glob_(i)*) quantifies the efficiency of parallel information transfer between that node and the network at large:
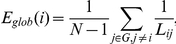
where *N* is the total number of nodes in the network and *L_ij_* is the shortest weighted path linking nodes *i* and *j*. Shortest paths are calculated as 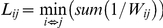, where 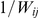 represents the functional distance between pairs of nodes (i.e., higher connection weights correspond to shorter functional distances; see [26]). High global efficiency thus indicates short average path lengths connecting a node to the rest of the network.

The local efficiency of an individual node *i* (*E_loc_(i)*) defines the efficiency of information transfer within the subgraph *G_i_* of nodes directly connected to node *i*:
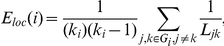
where the degree *k_i_* equals the number of nodes directly neighboring node *i*. This measure represents the speed of processing within the immediate functional vicinity of a node, with high local efficiency indicating efficient local functional organization [82]. Average network measures of global and local efficiency (*E_glob_* and *E_loc_*) may then be obtained by averaging the values across all individual nodes. Small-world networks possess *E_glob_* approaching (but slightly less than) that of comparable random networks (i.e., 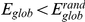), and *E_loc_* greater than random networks (i.e., 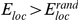). Since differences in mean connection strengths affect the values of *E_glob_* and *E_loc_*, we computed normalized 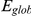 (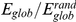) and 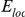 (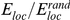) by dividing the network properties by the average values obtained in 50 randomized surrogate networks of conserved size and connectivity distribution [83]. Similarly, normalized measures were computed in individual cortical nodes by dividing the nodal efficiency measures by the network average *E_glob_* and *E_loc_* values in the randomized networks. To reduce the number of statistical comparisons, we computed summary measures by averaging the normalized *E_glob_* and *E_loc_* values across the entire network cost curves (for both the nodal and network average measures).

### Effects of age and task condition on network properties

We compared the cost-averaged network measures (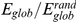 and 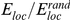) between younger and older adults and across task conditions using a repeated-measures, mixed-effects ANOVA model with task condition as a within-subjects factor and group as a between-subjects factor. In addition, we repeated this analysis for the cost-averaged 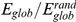 and 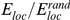 values within individual nodes to identify the cortical regions exhibiting altered network properties across subject groups and listening conditions.

### Network properties and mean cortical thickness

To test for a possible relationship between gross neuroanatomy and functional network properties, we obtained measures of cortical thickness for each subject using FreeSurfer [84], and computed Pearson's correlations between mean cortical thickness and the cost-averaged network measures (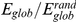 and 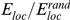).

## Supporting Information

Figure S1 **Comparison of whole-cortex global (** ***E_glob_*** **) and local (** ***E_loc_*** **) efficiency for cortical networks after regressing out performance (task accuracy) effects.** Error bars indicate the standard error of the mean. (O, older adults; Y, younger adults; Q, quiet listening condition; N, noisy listening condition)
(JPG)

Figure S2
**Aging and listening condition effects on whole-cortex network measures after regressing out performance (task accuracy) effects.** Summary values of 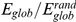 and 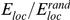 were obtained by averaging the measures across the entire cost curves for each subject and condition. Box plots indicate median, interquartile range, and minimum and maximum values of network measures across all subjects for each age group and listening condition.(JPG)

Methods S1 (DOC)

Table S1 (DOC)

Table S2 (DOC)
