# Supplementary figures and images for: Large-Scale Cortical Functional Organization and Speech Perception across the Lifespan
Source: PLoS One. 2011 Jan 31;6(1):e16510. doi: 10.1371/journal.pone.0016510 (PMC3031590; doi:10.1371/journal.pone.0016510)

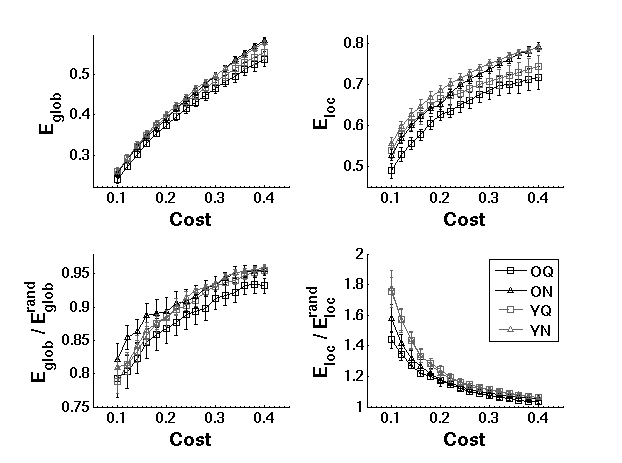

Supplement: Figure S1 — Comparison of whole-cortex global ( Eglob ) and local ( Eloc ) efficiency for cortical networks after regressing out performance (task accuracy) effects. Error bars indicate the standard error of the mean. (O, older adults; Y, younger adults; Q, quiet listening condition; N, noisy listening condition) (JPG) [file pone.0016510.s001.jpg]

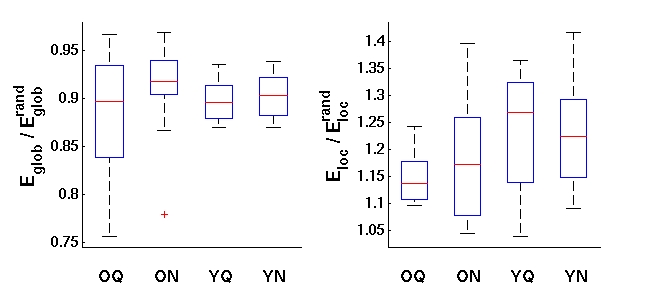

Supplement: Figure S2 — Aging and listening condition effects on whole-cortex network measures after regressing out performance (task accuracy) effects. Summary values of and were obtained by averaging the measures across the entire cost curves for each subject and condition. Box plots indicate median, interquartile range, and minimum and maximum values of network measures across all subjects for each age group and listening condition. (JPG) [file pone.0016510.s002.jpg]
