# Supplementary material for: Large-Scale Cortical Functional Organization and Speech Perception across the Lifespan
Source: PLoS One. 2011 Jan 31;6(1):e16510. doi: 10.1371/journal.pone.0016510 (PMC3031590; doi:10.1371/journal.pone.0016510)
Supplement: Methods S1 — (DOC) [file pone.0016510.s003.doc]

**Supplementary Methods**

*Cortical surface-based analysis*

In this study, we constructed high-resolution cortical surface maps for each individual subject using Freesurfer. In brief, the Freesurfer pipeline performs motion correction on the MRI T1 (structural) images, automatically removes non-brain tissues [1], transforms volumetric data to a common atlas, performs intensity normalization and topology correction [2-5], and defines the boundaries of the grey/white and pial surfaces [6-8].

Freesurfer also has the capability to parcellate the cerebral cortex based on arbitrary maps defined on a standard template. This process involves inflation of the cortical surfaces [9] and registration to a standard spherical atlas based on individual cortical folding patterns, which ensures topological alignment of cortical areas across subjects [10]. In this analysis, we used a version of the Desikan-Killiany cortical atlas [11] that parcellates the cerebral cortex into 33 cortical regions per hemisphere (see Figure 1, Main Text, for a visualization of this parcellation scheme).

Next, we used AFNI software [12] to map functional data from the native volumetric space of each subject (3.4375 mm x 3.4375 mm x 3 mm voxel resolution) directly onto the aligned cortical surface maps. Functional time courses were projected onto each surface vertex on the cortical surface meshes by interpolating between matching vertices on the grey/white and pial surfaces. For each vertex, 15 equally-spaced coordinates were sampled between the grey/white and pial surfaces, and data were projected by averaging across the unique 3D voxels overlapping these coordinates. In this fashion, surface vertices contained data only from voxels lying within the cortical grey matter. Subsequently, the projected functional data were averaged across all surface vertices in each of the 66 cortical regions of interest to extract regional time courses for the graph analysis.

*References*

1. Segonne F, Dale AM, Busa E, Glessner M, Salat D, et al. (2004) A hybrid approach to the skull stripping problem in MRI. Neuroimage 22: 1060-1075.

2. Sled JG, Zijdenbos AP, Evans AC (1998) A nonparametric method for automatic correction of intensity nonuniformity in MRI data. IEEE Trans Med Imaging 17: 87-97.

3. Fischl B, Liu A, Dale AM (2001) Automated manifold surgery: constructing geometrically accurate and topologically correct models of the human cerebral cortex. IEEE Trans Med Imaging 20: 70-80.

4. Fischl B, Salat DH, van der Kouwe AJ, Makris N, Segonne F, et al. (2004) Sequence-independent segmentation of magnetic resonance images. Neuroimage 23 Suppl 1: S69-84.

5. Segonne F, Pacheco J, Fischl B (2007) Geometrically accurate topology-correction of cortical surfaces using nonseparating loops. IEEE Trans Med Imaging 26: 518-529.

6. Dale AM, Sereno MI (1993) Improved Localization of Cortical Activity by Combining Eeg and Meg with Mri Cortical Surface Reconstruction - a Linear-Approach. Journal of Cognitive Neuroscience 5: 162-176.

7. Dale AM, Fischl B, Sereno MI (1999) Cortical surface-based analysis - I. Segmentation and surface reconstruction. Neuroimage 9: 179-194.

8. Fischl B, Dale AM (2000) Measuring the thickness of the human cerebral cortex from magnetic resonance images. Proc Natl Acad Sci U S A 97: 11050-11055.

9. Fischl B, Sereno MI, Dale AM (1999) Cortical surface-based analysis. II: Inflation, flattening, and a surface-based coordinate system. Neuroimage 9: 195-207.

10. Fischl B, Sereno MI, Tootell RB, Dale AM (1999) High-resolution intersubject averaging and a coordinate system for the cortical surface. Hum Brain Mapp 8: 272-284.

11. Desikan RS, Segonne F, Fischl B, Quinn BT, Dickerson BC, et al. (2006) An automated labeling system for subdividing the human cerebral cortex on MRI scans into gyral based regions of interest. Neuroimage 31: 968-980.

12. Cox RW (1996) AFNI: Software for analysis and visualization of functional magnetic resonance neuroimages. Computers and Biomedical Research 29: 162-173.
