# Supplementary material for: Large-Scale Cortical Functional Organization and Speech Perception across the Lifespan
Source: PLoS One. 2011 Jan 31;6(1):e16510. doi: 10.1371/journal.pone.0016510 (PMC3031590; doi:10.1371/journal.pone.0016510)
Supplement: Table S1 — (DOC) [file pone.0016510.s004.doc]

**Table S1. Effects of age group and listening condition on whole-cortex network measures after regressing out performance (task accuracy) effects.**

| $E_{glob}/E_{glob}^{rand}$ | | | $E_{loc}/E_{loc}^{rand}$ | | |
| --- | --- | --- | --- | --- | --- |
| **Effect** | **F(1,22)** | **Sig.** | **Effect** | **F(1,22)** | **Sig.** |
| **Group** | 0.075 | .786 | **Group** | 4.983 | .036 |
| **Condition** | 4.991 | .036 | **Condition** | 0.312 | .582 |
| **Interaction** | 1.942 | .177 | **Interaction** | 0.460 | .505 |
