# Supplementary material for: Large-Scale Cortical Functional Organization and Speech Perception across the Lifespan
Source: PLoS One. 2011 Jan 31;6(1):e16510. doi: 10.1371/journal.pone.0016510 (PMC3031590; doi:10.1371/journal.pone.0016510)
Supplement: Table S2 — (DOC) [file pone.0016510.s005.doc]

**Table S2. Effects of age group and listening condition on regional network measures after regressing out performance (task accuracy) effects.**

| $E_{glob}/E_{glob}^{rand}$ | | | | $E_{loc}/E_{loc}^{rand}$ | | | |
| --- | --- | --- | --- | --- | --- | --- | --- |
| **Group effect** | **Effect** | **F(1,22)** | **Sig.** | **Group effect** | **Effect** | **F(1,22)** | **Sig.** |
| Left IT | Y > O | 6.26 | .0203 | Left SP | Y > O | 4.92 | .0372 |
| Left TT | Y > O | 4.48 | .0459 | Left MOF | Y > O | 7.43 | .0123 |
| Right IT | Y > O | 5.40 | .0298 | Left RAC | Y > O | 6.39 | .0191 |
| Right LOF | Y > O | 17.06 | .0004 | Left ST | Y > O | 4.37 | .0484 |
| Right MT | Y > O | 5.20 | .0326 | Right BSTS | Y > O | 6.09 | .0219 |
| Right ISTC | O > Y | 5.71 | .0258 | Right IT | Y > O | 4.97 | .0363 |
|  |  |  |  | Right LOF | Y > O | 20.24 | .0002 |
| **Condition** | **Effect** | **F(1,22)** | **Sig.** | Right RAC | Y > O | 10.08 | .0044 |
| Left BSTS | N > Q | 5.13 | .0337 |  |  |  |  |
| Left IP | N > Q | 4.56 | .0441 | **Condition** | **Effect** | **F(1,22)** | **Sig.** |
| Left PCUN | N > Q | 13.36 | .0014 | Left SP | N > Q | 4.87 | .0381 |
| Left SP | N > Q | 4.56 | .0441 | Left MOF | Q > N | 8.06 | .0096 |
| Left MOF | Q > N | 5.73 | .0256 | Right MT | N > Q | 6.98 | .0149 |
| Right BSTS | N > Q | 4.42 | .0473 | Right ST | N > Q | 5.73 | .0256 |
| Right MT | N > Q | 9.79 | .0049 | Right TT | N > Q | 5.47 | .0288 |
| Right PSTC | N > Q | 5.35 | .0304 | Right ENT | Q > N | 7.05 | .0144 |
| Right PCUN | N > Q | 14.93 | .0008 | Right FP | Q > N | 5.98 | .0229 |
| Right SP | N > Q | 12.80 | .0017 | Right LOF | Q > N | 11.14 | .0030 |
| Right ST | N > Q | 8.04 | .0096 | Right TP | Q > N | 14.78 | .0009 |
| Right SMAR | N > Q | 5.32 | .0308 |  |  |  |  |
| Right ENT | Q > N | 8.15 | .0092 | **Interaction** | **Effect** | **F(1,22)** | **Sig.** |
| Right LOF | Q > N | 5.34 | .0306 | Left PHG | O(N>Q) > Y(N>Q) | 4.82 | .0389 |
| Right FP | Q > N | 7.05 | .0145 | Right MOF | O(N>Q) > Y(N>Q) | 4.56 | .0442 |
| Right TP | Q > N | 17.75 | .0004 |  |  |  |  |
|  |  |  |  |  |  |  |  |
| **Interaction** | **Effect** | **F(1,22)** | **Sig.** |  |  |  |  |
| Left ISTC | O(N>Q) > Y(N>Q) | 5.80 | .0248 |  |  |  |  |
| Right ISTC | O(N>Q) > Y(N>Q) | 4.32 | .0496 |  |  |  |  |
